# Prediction of sex-determination mechanisms in avian primordial germ cells using RNA-seq analysis

**DOI:** 10.1038/s41598-022-17726-7

**Published:** 2022-08-17

**Authors:** Kennosuke Ichikawa, Yoshiaki Nakamura, Hidemasa Bono, Ryo Ezaki, Mei Matsuzaki, Hiroyuki Horiuchi

**Affiliations:** 1 Genome Editing Innovation Center, Hiroshima University, 3-10-23 Kagamiyama, Higashi-Hiroshima, Hiroshima, 739-0046 Japan; 2 Graduate School of Integrated Sciences for Life, Hiroshima University, 1-4-4 Kagamiyama, Higashi-Hiroshima, Hiroshima, 739-8528 Japan

**Keywords:** Germline development, Differentiation

## Abstract

In birds, sex is determined through cell-autonomous mechanisms and various factors, such as the dosage of DMRT1. While the sex-determination mechanism in gonads is well known, the mechanism in germ cells remains unclear. In this study, we explored the gene expression profiles of male and female primordial germ cells (PGCs) during embryogenesis in chickens to predict the mechanism underlying sex determination. Male and female PGCs were isolated from blood and gonads with a purity > 96% using flow cytometry and analyzed using RNA-seq. Prior to settlement in the gonads, female circulating PGCs (cPGCs) obtained from blood displayed sex-biased expression. Gonadal PGCs (gPGCs) also exhibited sex-biased expression, and the number of female-biased genes detected was higher than that of male-biased genes. The female-biased genes in gPGCs were enriched in some metabolic processes. To reveal the mechanisms underlying the transcriptional regulation of female-biased genes in gPGCs, we performed stimulation tests. Retinoic acid stimulation of cultured gPGCs derived from male embryos resulted in the upregulation of several female-biased genes. Overall, our results suggest that sex determination in avian PGCs involves aspects of both cell-autonomous and somatic-cell regulation. Moreover, it appears that sex determination occurs earlier in females than in males.

## Introduction

Birds have unique mechanisms of sex determination. Mammals, which have an XX/XY sex chromosome system, determine their gonadal sex depending on the transient action of a Y-chromosome–linked master gene, *Sex-determining region Y* (*SRY*)^1^. In the case of birds, which have a ZZ/ZW sex chromosome system, the dosage of *doublesex and mab-3 related transcription factor 1* (*DMRT1*) on the Z chromosome is key to gonadal sex determination^2–4^. The expression of *DMRT1* is restricted to the developing gonads of both sexes but is more highly expressed in males than in females owing to the fact that this gene is Z linked. Avian somatic cells possess an inherent sex identity, and gonadal development and sexual phenotype are largely cell autonomous^5^. Nevertheless, sex hormones also primarily regulate sexual phenotypes, and therefore, sex-reversal, a characteristic feature in fish, is also observed in birds. Investigation of the sex-determination mechanism in birds will provide an insight into the evolution of vertebrate sex-determining mechanisms.

Little is known about the sex-determining mechanism in avian germ cells, unlike the mechanism in somatic cells. The mechanisms underlying sex determination in germ cells generally have received increasing attention in the past decade. In vertebrates, *forkhead box L3* (*FOXL3*) was the first identified gene associated with sex determination in germ cells in medaka (*Oryzias latipes*)^6^. Development of functional sperm in the ovary of *FOXL3-*knockout female medaka indicated that the germline possesses a sex-determination mechanism different from that present in the gonads in at least some vertebrates. However, in chickens, the *FOXL3*-like gene is temporally expressed in oogonia, which develop after sex determination in germ cells^7^. Therefore, the sperm–egg fate decision via *FOXL3* is not conserved in chickens. To comprehensively understand the mechanism underlying avian sex determination, avian-specific sperm-egg fate determinants must be revealed.

To elucidate the sex-determination mechanism in avian germ cells, gene profiling in primordial germ cells (PGCs), the first germ cell population established during early development, obtained from various developmental stages, is required. Sex differentiation of germ cells is generally induced by the surrounding gonadal somatic cells. As gonadal masculinization-related genes, *DMRT1* and *hemogen* (*HEMGN*), or feminization-related genes, *forkhead box L2* (*FOXL2*) and *aromatase*, begin to be expressed in chicken embryos incubated for 4.5–5.7 days (E4.5–5.7)^3,8,9^, sex differentiation in gonadal PGCs (gPGCs) might also be induced from this stage onward. Interestingly, cell-autonomous sex determination of chicken PGCs was also suggested before the cells settled into the gonads. Unlike the PGCs in other vertebrates, avian PGCs are transported to the genital ridge through blood circulation. Although the PGC precursor cells transplanted into chicken embryos of the opposite sex at the blastodermal stage could differentiate into functional gametes^10^, circulating PGCs (cPGCs) transplanted into the bloodstream barely differentiate into functional gametes^11,12^. Additionally, the number of PGCs located in the intermediate mesoderm and the capacity of cultured PGCs derived from embryonic blood to uptake proteins differ between males and females^13,14^. Although a recent study showed that donor PGC-derived offspring could be produced using genetically infertile chickens of the opposite sex as recipients^15^, the sex determination mechanism of PGCs remains controversial. Therefore, to reveal this mechanism in avian PGCs, the effects of the surrounding gonadal cells and cell-autonomous factors must be considered.

In order to investigate the mechanisms underlying sex determination in avian germ cells, we purified male and female PGCs from blood (E2.5; Hamburger and Hamilton stage^16^ (HH) 17) and gonads (E4.5 and E6.5; HH25-26 and HH30, respectively) using fluorescence-activated cell sorting (FACS). Gene expression profiles of PGCs at each developmental stage for each sex were determined using RNA-seq analysis. Then, the sex-determination mechanism of PGCs was predicted using bioinformatic analysis. To evaluate the prediction, male PGCs were stimulated with retinoic acid in vitro*,* and the changes in gene expression were examined.

## Results

### Purification of PGCs using FACS

**Figure 1:**
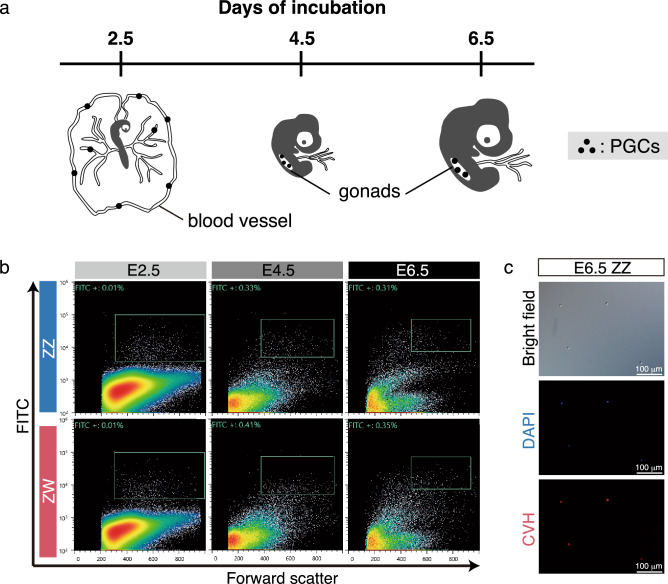
Purification of PGCs from early chick embryos. (**a**) A schematic illustration of localization of PGCs during embryogenesis. (**b**) Dot-plots of forward scatter (x-axis) versus green-fluorescence (y-axis). SSEA-1 positive cells were sorted in each stage and sex (boxes). (**c**) Immunofluorescence of sorted cells derived from E6.5 male embryos using anti-CVH monoclonal antibody. Counter staining was carried out with DAPI.

FACS was used to sort male and female PGCs from the blood of E2.5 embryos and gonads of E4.5 and E6.5 embryos, respectively (Fig. 1a). Herein, a monoclonal antibody against SSEA-1, a cell surface marker of chicken PGCs found during early development^17^, was used to harvest PGCs. Figure 1b shows the cell sorting patterns obtained for each sample.

After sorting, the purity of PGCs was evaluated with immunofluorescence using a monoclonal antibody against chicken VASA homologue (CVH), a chicken pan-germ cell marker^18^. Expression of CVH was observed in the cytoplasm, and the purity of CVH-positive PGCs was > 96% irrespective of the developmental stage and sex (Fig. 1c; Table1). RNA-seq analysis was performed using the sorted PGCs under these conditions.

**Table 1:** Purities of sorted PGCs in each stage and sex.

Developmental stage	Sex	Analyzed cells	CVH + cells	Purity (%)	Donors (n)
E2.5	ZZ	189	186	98.4	22
	ZW	333	326	97.9	30
E4.5	ZZ	89	88	98.9	20
	ZW	72	71	98.6	22
E6.5	ZZ	198	190	96.0	22
	ZW	172	168	97.7	20

Data from two independent experiments were collected in each stage and sex.

### Detection of sex-biased genes using RNA-seq analysis

To predict the sex-determination mechanism in chicken PGCs, RNA-seq analysis was performed using sorted PGCs. Approximately 15.7 million total reads per sample were obtained (Supplementary Table S1). On average, nearly 88% of these reads were uniquely mapped to the GRCg6a reference genome.

**Figure 2:**
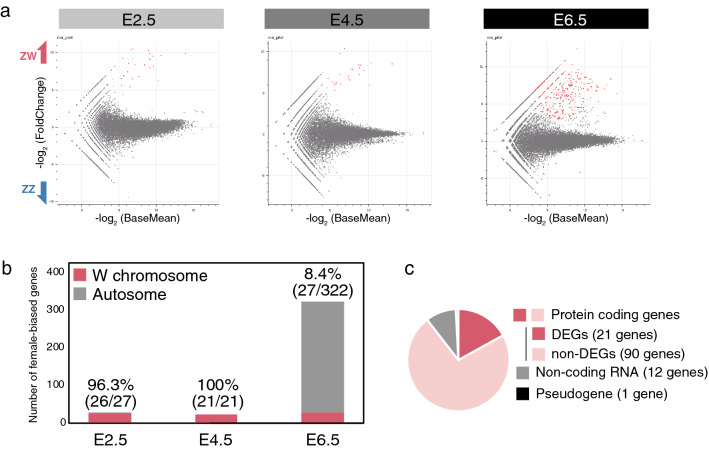
Detection and characterization of sex-biased genes during embryogenesis. (**a**) MA-plots of males versus females at each stage. Female-biased genes (log_2_ (FC) ≥ 3, Padj < 0.05) are shown in red. Male-biased genes (log_2_ (FC) ≤  − 3, Padj < 0.05) are shown in blue. (**b**) Chromosomal localization of the female-biased genes in each stage. Y-axis indicates the number of genes. The ratio of W-linked genes to whole sex-biased genes is presented on each bar. (**c**) Distribution of W-linked genes detected as female-biased genes in common to each stage.

Subsequently, we identified differentially expressed genes (DEGs) (|log_2_ (FC)|≥ 3, adjusted *p*-value (Padj) < 0.05) as sex-biased genes at each developmental stage. Overall, while the number of sex-biased genes was very low in E2.5 (31 genes) and E4.5 (22 genes) embryos, it was dramatically increased in E6.5 embryos (336 genes) (Fig. 2a, Table 2). All sex-biased genes detected in this study are listed in supplementary Table S2. The number of female-biased genes was higher than that of male-biased genes at every stage. Alterations in gene expression profiles in female PGCs were observed prior to those in male PGCs.

**Table 2:** Number of sex-biased genes.

Developmental stage	Male-biased genes	Female-biased genes	Total sex-biased genes
E2.5	4	27	31
E4.5	1	21	22
E6.5	14	322	336

Chromosomal localization of female-biased genes was also analyzed at each stage. The vast majority of female-biased genes in both E2.5 and E4.5 embryos were localized on the W chromosome (Fig. 2b). In contrast, the number of female-biased genes were dramatically increased in E6.5 embryos, and 91.6% of these genes (295/322) were autosomal. Twenty-eight genes were investigated as W chromosomal female-biased genes in E2.5, E4.5, and E6.5, and 21 genes were commonly detected. These detected genes were protein-coding genes, and corresponded to 18.9% (21/111) of those linked to W chromosome (Fig. 2c). The detected common female-biased genes are listed in supplementary Table S3 with functional domains predicted using Pfam^19^, a database of protein families. The gene names were annotated to refer to a previous study^20^, which globally characterized chick W-linked genes. The functional predictions revealed that several genes possess a DNA-binding domain. These were *SMAD2W* (PF03165), *SMAD7B* (PF03165), *SUB1W* (PF02229), *KCMF1W* (PF00569), and *MIER3W* (PF01448 and PF00249). Additionally, a chromatin remodeling-related domain (PF00385) was detected in *CHD1W*. Overall, several W-linked genes were expressed and maintained in PGCs in a cell-autonomous manner, and some possessed functional domains that could regulate transcription mechanisms.

### Enrichment analysis of female-biased genes in gPGCs derived from E6.5 embryos

**Figure 3:**
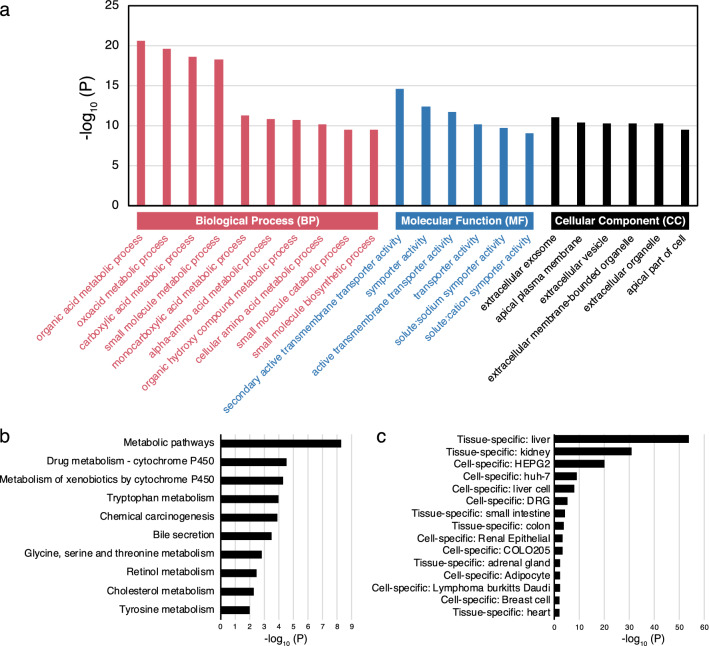
Classification of female-biased genes in E6.5 embryos. (**a**) The most enriched terms observed through gene ontology (GO) analysis. Three main categories, biological process (BP), molecular function (MF), and cellular component (CC), are denoted using red, blue, and black colors, respectively. (**b**) The results of Kyoto Encyclopedia of Genes and Genomes (KEGG)^21^ pathway analysis. (**c**) The results of tissue- or cell- specificity.

Enrichment analysis was performed to characterize the features of female-biased genes detected in E6.5. First, we performed gene ontology (GO) analysis. Figure 3a shows highly enriched GO terms, classified as biological process (BP), molecular function (MF), or cellular component (CC). Some metabolic processes were highly enriched in the BPs. In particular, organic acid, oxoacid, carboxylic acid, and small molecule metabolic processes were detected as major terms in GO analysis of female-biased genes in E6.5. In contrast, the enriched MFs were related to transporter or symporter activity, and the CCs were extracellular or apical parts. We then performed Kyoto encyclopedia of genes and genomes (KEGG) ^21^ pathway analysis. KEGG pathway analysis also revealed enrichment of some metabolic pathways (Fig. 3b). Finally, tissue- or cell-specificity of female-biased genes was also characterized using the PaGenbase database^22^. Interestingly, many female-biased genes were annotated as specific genes in the liver, kidney, and liver-derived cell lines (HEPG2 and huh-7) (Fig. 3c). All detected terms and enriched genes in each term are listed in supplementary Table S4.

**Figure 4:**
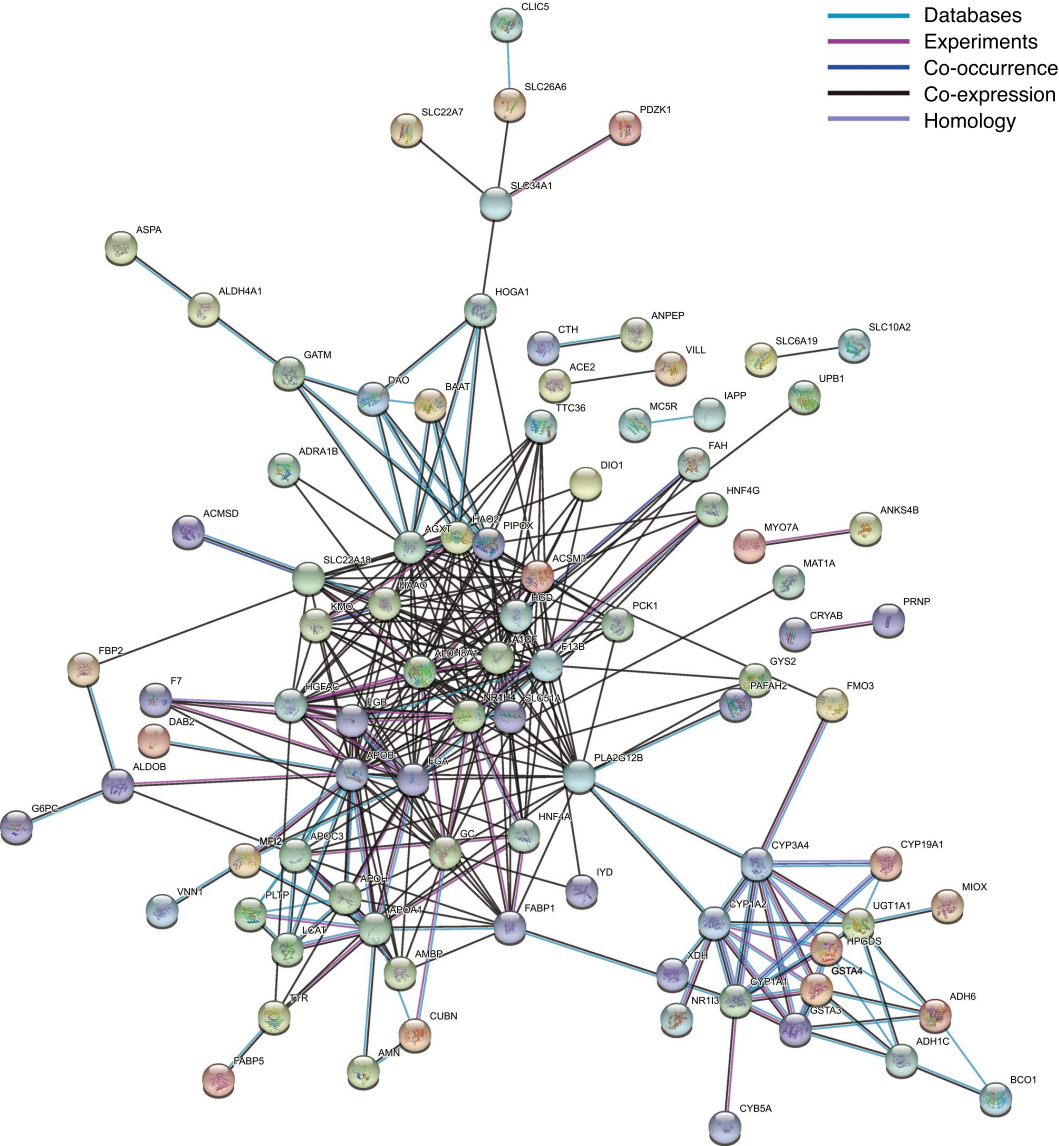
The protein–protein interaction network of female-biased genes in E6.5 chicken embryos. Nodes and edges represent protein and interaction, respectively. Different colors of edges correspond to different associations.

To reveal the protein–protein interaction (PPI) network of female-biased genes in E6.5, we further analyzed the STRING network (Fig. 4). Subsequently, 204 and 326 nodes and edges were obtained, respectively, and the average node degree was 3.2. The average local clustering coefficient was 0.315. The PPI enrichment p-value was lower than 1.0e-16, and this network thus significantly interacted rather than being selected at random.

### Confirmation of the RNA-seq results using RT-qPCR

We examined the expression levels of several genes, including *regucalcin* (*RGN*), *cytochrome b5 type A* (*CYB5A*), *UDP glucuronosyltransferase family 1 member A1* (*UGT1A1*), *apolipoprotein A1* (*APOA1*), *hematopoietic prostaglandin D synthase* (*HPGDS*), *glycine amidinotransferase* (*GATM*), *aldolase, fructose-bisphosphate B* (*ALDOB*), and *beta-ureidopropionase* 1 (*UPB1*). These genes were included in all four major GO terms in the GO analysis: organic acid metabolic process, oxoacid metabolic process, carboxylic acid metabolic process, and small molecule metabolic process. Moreover, *RGN*, *CYB5A*, *UGT1A1*, *APOA1*, *ALDOB*, and *UPB1* were also enriched as liver-characteristic genes. Although *APOA1* and *ALDOB* were also highly expressed in gPGCs derived from E4.5 female embryos, high expression levels of all genes were commonly observed in gPGCs derived from E6.5 female embryos (Supplementary Fig. S1). The results of RT-qPCR corresponded to those of RNA-seq in terms of the upregulation of the candidate genes in E6.5 female gPGCs.

### RA stimulation of gPGCs in vitro

**Figure 5:**
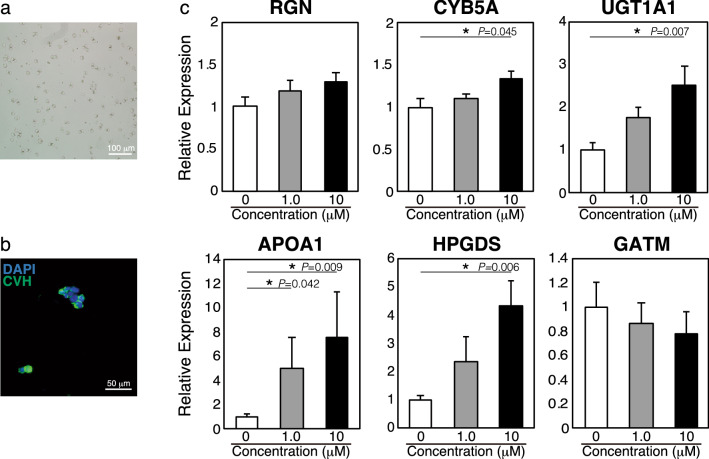
Stimulation test using RA. (**a**) The cultured and proliferated gPGCs derived from E6.5 male chicken embryos. (**b**) Immunofluorescence with an anti-CVH antibody. Counter staining was carried out with DAPI. (**c**) Expression analysis of the female-biased genes under RA stimulation. The x-axis indicates the concentration of RA. The 2^−ΔΔCt^ method was used for the calculation of relative expression levels, which were normalized using expression levels of *β-actin*. Error bars indicate SE of the mean of relative expression levels in independently cultured gPGCs derived from five individuals (*n* = 5). Significance was evaluated using Dunnett’s test. Asterisks represent significant differences (*P < 0.05).

To investigate the factors involved in the upregulation of the female-biased genes in gPGCs derived from E6.5 female embryos, a stimulation test using cultured gPGCs was performed. We focused on retinoic acid (RA) as a candidate factor involved in the upregulation of these genes. RA directly induces meiosis in female germ cells during embryogenesis, and thus RA is a significant factor in the feminization of PGCs. Here, gPGCs derived from E6.5 male embryos were cultured and proliferated (Fig. 5a). Immunofluorescence revealed that the cultured gPGCs expressed CVH (Fig. 5b). Moreover, the expression of SSEA-1 was maintained in cultured gPGCs (data not shown). A stimulation test using RA was then conducted. The results of the expression analysis of the female-biased genes using RT-qPCR are shown in Fig. 5c. Several female-biased genes, including *CYB5A*, *UGT1A1*, *APOA1*, and *HPGDS*, showed upregulated expression after RA stimulation in a dose-dependent manner. Although no significance was achieved, the expression of *RGN* was also induced. In contrast, the expression of *GATM* was decreased rather than increased, but no significant difference was observed. *ALDOB* and *UPB1* were not detected in this test, irrespective of RA stimulation.

Additionally, the expression of RA receptors, *retinoic acid receptor alpha* (*RARα*), *beta* (*RARβ*), and *gamma* (*RARγ*) were analyzed using RT-PCR to evaluate the responsibility against RA of the cultured PGCs. Although our RNA-seq results showed prominent expression of *RARβ* during these receptors (Supplementary Fig. S2a), all receptors were expressed in the cultured PGCs (Supplementary Fig. S2b). Therefore, while the expression patterns of the RA receptors might not be conserved between the PGCs in vivo and the cultured condition, the responsibility against RA was suggested.

We further analyzed the expression of *stimulated by retinoic acid 8* (*STRA8*), a meiotic marker^23,24^, to confirm whether meiosis was induced in this stimulation test. The *STRA8* expression was unchanged under the conducted conditions (Supplementary Fig. S3). Therefore, meiosis could not be induced in the current stimulation test. Nevertheless, it is interesting that the expression of several female-biased genes was upregulated by the single stimulus of RA in the male-derived cultured PGCs.

Overall, RA can enhance the upregulation of several female-biased genes in male-derived gPGCs. Furthermore, other factors, including the cell-autonomous differentiation, might be involved in the feminization of gPGCs.

## Discussion

The objective of this study was to investigate the mechanism underlying sex determination in avian PGCs. Therefore, female-specific alterations in gene expression profiles were investigated and identified. It was observed that sex determination may occur earlier in females than in males and involve cell-autonomous and RA regulation aspects. A very recent study demonstrated the sex-differences between early embryonic PGCs (mix of E2.5 to E8.0) using transcriptome analysis^25^. However, this study did not demonstrate the timing and factors underlying sex determination. To the best of our knowledge, the current report is the first to predict the sex-determination mechanism by comparing the gene expression profiles of avian PGCs at each embryonic stage as well as by using a stimulation test.

The number of female-biased genes was higher than that of male-biased genes during early development (Fig. 2 and Table 2). Thus, the findings of this study provide an insight into the mechanism underlying female-specific sex differentiation in terms of the gene expression profiles. Interestingly, avian gonadal sex determination has the opposite effect. *DMRT1*, a candidate gene in avian gonadal sex determination that is present on the Z chromosome, is expressed in both sexes from approximately E4.5 onward. In birds, dosage compensation for the sex chromosome is incomplete^26^, and the expression levels of *DMRT1* in males are also higher than those in females, as *DMRT1* is a Z-linked gene^27^. The strong expression of *DMRT1* induces the upregulation of masculinization-related genes, such as *HEMGN* and *SOX9*, a phenomenon that results in testicular development^3^. In contrast, low expression of *DMRT1* in females results in ovarian development, including upregulation of *FOXL2*^2,4^, a transcription factor that prevents testicular development^28^. Therefore, gonadal sex determination is likely triggered by male-specific gene expression. Indeed, analysis of the gene expression profiles of chick gonads in E4.5 revealed that the number of male-biased genes was higher than that of female-biased genes^29^. Additionally, Ayers and colleagues also reported that the increase in the number of male-biased genes was larger than that of female-biased genes from E4.5 to E6.0^30^. Overall, although avian gonadal sex determination is triggered by the expression of male-specific genes, our findings suggests that in avian PGCs, sex determination proceeds predominantly in females.

We detected 21 W-linked female-biased genes, which were consistently expressed from E2.5 to E6.5 (Fig. 2b,c and supplementary Table S3). As the W-linked genes were upregulated in female cPGCs, these genes could be expressed independently of the signals from gonadal somatic cells. Among the 21 W-linked genes, some candidates were identified as potential regulators in the establishment of sexual identity. *SMAD2W* and *SMAD7B* possess the SMAD domain, which is conserved in the SMAD family. The proteins belonging to the SMAD family are highly phosphorylated in chicken PGCs, and SMAD signaling activation is essential for the in vitro proliferation of PGCs^31^. The *SMAD7B* gene was cloned in a previous study^32^. However, the functions in sex determination of SMAD7B and SMAD2W are undetermined. Conversely, HINTW is a well-analyzed W-linked candidate exhibiting characteristic strong expression patterns in female embryos^33,34^. Although HINTW overexpression did not affect gonadal masculinization^35^, its function in sex determination in PGCs remains unclear. Additionally, several candidates that potentially regulate the expression of other genes were also identified in this study. In future studies, functional analysis of W-linked genes in PGCs is warranted.

Several previous studies have also demonstrated sexual differences in chicken PGCs at the pre-gonadal stage. First, sexual bipotency was lost in cPGCs. Compared to PGC precursor cells, which are included in blastodermal cells, cPGCs hardly differentiate into gametes in the opposite sex gonads upon transplantation^10–12^. Proliferation activity of PGCs might also be different between males and females. The number of female PGCs concentrated in the intermediate mesoderm in E2.5 embryos was significantly higher than that of male PGCs^13^. Our study provides new insights into some candidates that may contribute to the gonadal differentiation-independent sex determination of PGCs.

In E6.5 embryos, female PGCs strongly showed sex-biased gene expression profiles with expressions of several metabolic processes related genes (Figs. 3, 4, and Supplementary Fig. S1). After gonadal sex determination, chick germ cells showed sex-specific developmental patterns. While male germ cells barely proliferate during embryonic testicular development, female germ cells exhibit high proliferating activity from at least E9.0^36,37^. Then, female germ cells undergo meiosis in E15.5. The metabolic processes of female PGCs at E6.5 may contribute to the female germ cell-specific proliferation activity. Conversely, we could not detect well-known processes or pathways related to sex determination and gametogenesis in this stage. Thus, we could not identify any direct gene or process determining sex in the chicken PGC. However, since not all of the genes annotated to date can explain the sex determination mechanism in birds, the findings of this study provide new insights that are needed to elucidate this mechanism.

Previous studies have demonstrated the activation of several metabolic processes in PGCs. Prostaglandin D_2_ (PGD_2_), which is synthesized by HPGDS and lipocalin-type prostaglandin D synthase (LPGDS), is produced in murine testicular PGCs and is related to sex differentiation in these cells^38^. Calcium (Ca^2+^) homeostasis also contributes to gametogenesis. RGN is a Ca^2+^-binding protein and is involved in the regulation of several enzymatic activities, such as cell proliferation and apoptosis. Previously studied as a characteristic factor of the liver and kidney^39,40^, RGN is also expressed in mammalian testes, including germ cells, and is likely related to spermatogenesis^41,42^. Furthermore, since murine early embryonic PGCs exhibit high glycolytic activity^43^, the contribution of the glycolytic pathway, including activity of ALDOB, to the development of these pathways is likely. Additionally, gene profiling analyses comparing chick gPGCs and blastoderms under mixed-sex conditions revealed upregulation of *APOA1*, *RGN*, and nucleotide metabolism-related genes, including *UPB1*, in the gPGCs^44,45^. Our study provides new evidence that suggests that the activation of these metabolic process-related genes is a characteristic feature of female gPGCs, at least during the early developmental stage.

Although our result showed female-specific potential pathways and processes, Rengaraj et al. reported many male-biased genes via transcriptome analysis using PGCs collected from early embryos (E2.5-E8) in a very recent study^25^. These differences may be caused by molecular markers used in purifying the PGCs. We sorted the PGCs through FACS targeting SSEA-1, while Rengaraj et al. isolated PGCs using genome-edited chickens fluorescently labeled with deleted in azoospermia like (DAZL), a pan-germ cell marker. Although SSEA-1 has been used as a marker of the PGCs^17,46–48^, recent studies have showed the existence of SSEA-1-negative PGCs^49^. Thus, this can reflect the differences in gene expression profiles. Interestingly, while the SSEA-1-positive PGCs at least possess a germline transmission^47^, the effect of SSEA-1 expression on germ cell development remains unknown. Thus, we believe that the results of this study and the report of Rengaraj et al. will provide important insights into the association between SSEA-1 expression and sex determination.

DMRT1 plays an essential role in germ cell sex determination in tilapia^50^. Since DMRT1 significantly contributes to testis development in chickens^2–4^, that contribution to sex determination of PGCs was expected. However, this study provided no data suggesting the relationship between DMRT1 and the sex determination of PGCs. In a previous study, sex-biased expression of *DMRT1* was observed in the germ cells of female chick embryos at E16, which corresponds to the meiotic stage^25^. Therefore, DMRT1 may play an essential role in germ cell development even later than the embryonic stages we evaluated in this study.

Stimulation of male PGCs obtained from the gonads of E6.5 embryos with RA in vitro resulted in the upregulation of several female-biased genes (Fig. 5). RA is a major factor inducing feminization in embryonic germ cells. In female chick germ cells at E12.5, RA induces the expression of STRA8, resulting in meiosis at E15.5. In this study, *STRA8* expression was not induced in the stimulation test (Supplementary Fig. S3), suggesting that this test is insufficient in inducing meiosis in vitro. To induce meiosis in mammalian PGC-like cells (PGCLCs), it is necessary to differentiate the PGCLCs as late PGC-like features and stimulate them with RA as well as bone morphogenetic proteins (BMPs)^51^. Therefore, to induce meiosis in chick PGCs, further study will be needed to establish a novel culture condition. Nevertheless, our findings suggest that RA also regulates the upregulation of several female-biased genes in female PGCs at E6.5 before *STRA8* expression. To confirm this hypothesis, RA expression and function during gonadal sex determination should be analyzed in vivo in subsequent studies.

In this study, PGCs cultured and proliferated for two months were used owing to the limitated number of primary PGCs that can be harvested from an embryo. Therefore, the PGCs used in the stimulation test were maintained in an environment different from that required by PGCs in vivo. Nevertheless, in our previous study^52^, we have successfully produced offspring from PGCs cultured in the same condition. Further, the cultured PGCs and in vivo PGCs expressed CVH (Fig. 1c and 5b) and *RARβ* (Supplementary Fig. S2). While there was a difference in terms of the expression pattern of RA receptors in PGCs between in vitro and in vivo, we have considered that these cultured PGCs meet the minimum requirement for assessing the RA function.

RA-independent regulatory factors could also be involved in the feminization of chicken PGCs because RA had no effect on the expression of *GATM* and *RGN* (Fig. 5). As an RA-independent regulatory factor, sexual differences in DNA methylation patterns were predicted. A previous study demonstrated that the methylation in *GATM* gene is significantly higher in E6.0 male gPGCs than that in female gPGCs^53^. This is consistent with our findings that RA stimulation could not induce *GATM* expression in male gPGCs obtained from embryos at E6.5. Additionally, this also suggests cell-autonomous sex differentiation in PGCs after gonadal sex determination.

Overall, we successfully detected the sex-biased genes, mainly female-biased genes, in chick PGCs during early embryonic stages and predicted female-specific potential processes and pathways in the stages. However, whether the predicted processes and pathways contribute to the feminization of PGCs remain unclear. Therefore, further investigations are warranted to conclusively establish the functional correlations between the female-biased genes and the sex determination of PGCs. Since 2014, an effective strategy to produce genetically modified chickens using genome editing tools is being extensively used^54^. This protocol is also used for basic research regarding germ cell development^55^. Further studies and functional analyses of genetic modifications may identify the key molecule(s), which induce(s) feminization of chick PGCs, among the female-biased genes.

## Methods

### Experimental animals

Fertilized eggs of white Leghorn were purchased from Akita foods (Fukuyama, Japan) and used for PGC collection from blood and gonads. All animal care and use protocols in this study were conducted in accordance with the animal experimentation guidelines of the Hiroshima University Animal Research Committee (approved protocol ID: C21-45–2) and the ARRIVE guidelines.

### Sample preparation using flow cytometry

Male and female PGCs were isolated from the blood of E2.5 embryos and from the gonads of E4.5 and E6.5 embryos using FACS. Fertilized eggs were incubated at 37 °C under 50–60% relative humidity. Collected gonad cells were dissociated using TrypLE™ Express Enzyme (Thermo Fisher Scientific, Carlsbad, CA, USA). The gonad cells and blood cells were incubated with anti-stage-specific embryonic antigen-1 (SSEA-1) antibody (sc-2170; Santa Cruz Biotechnology, Dallas, TX, USA) diluted with wash buffer (0.5% BSA and 0.1% NaN_3_-PBS) at 1:100 for 1 h on ice. After washing with the washing buffer, the secondary antibody reaction was conducted using FITC Rat anti-mouse IgM antibody (553,408; BD Biosciences, Franklin Lakes, NJ, USA) at 1:200 dilution for 30 min on ice. After washing, the samples were incubated with propidium iodide to detect the dead cells. Then the SSEA-1 positive cells were sorted using Cell Sorter MA900 (Sony Biotechnology, San Jose, CA, USA). The sex of each embryo was confirmed using the patterns of *chromatin-helicase DNA-binding protein-1* (CHD1) fragment bands obtained through PCR^56^. Genomic DNA was purified from a small section of embryos using SimplePrep™ reagent for DNA (TaKaRa Bio, Kusatsu, Japan).

### Immunofluorescence

The purity of the sorted cells was confirmed using immunofluorescence with an anti-CVH mouse monoclonal antibody^57^. First, the sorted cells were adhered to slides with Smear Gell (GenoStaff, Tokyo, Japan) according to the manufacturer’s instructions. Subsequently, the adhered cells were fixed with 4% paraformaldehyde in PBS for 30 min at room temperature (approximetry 20 °C). After washing with 0.1% BSA-PBS, the adhered cells were incubated with 0.1% Tween-20. Subsequently, they were blocked with 3% BSA, and then the primary antibody reaction using hybridoma supernatant containing the anti-CVH antibody, which was diluted 1:1 with 2% BSA-PBS, was performed for 40 min at 37 °C. Next, the cells were washed, and the secondary antibody reaction was performed using goat anti-mouse IgG (H + L) (Alexa Fluor Plus 555; Invitrogen, Waltham, MA, USA) at 1:200 dilution with 1% BSA-PBS for 40 min at 37 °C. Finally, nuclei were stained with 4′,6-diamidino-2-phenylindole (DAPI) in VECTASHIELD Mounting Medium (Vector Laboratories Inc., Burlingame, CA, USA) after washing. They were observed under a fluorescence microscope (BX53; Olympus, Tokyo, Japan) and photographed with a DP74 camera (Olympus, Tokyo, Japan).

Furthermore, cultured male PGCs obtained from the gonads of E6.5 embryos were centrifuged and fixed in 4% paraformaldehyde prepared in PBS for 30 min at room temperature (approximetry 20 °C) after washing. Immunofluorescence was performed using the same procedure as described above until primary antibody probing. After washing, incubation with the secondary antibody was performed using goat anti-mouse IgG (H + L) (Alexa Fluor Plus 488; Invitrogen) under the same conditions as described above. The samples were then washed and mounted on glass slides using VECTASHIELD Mounting Medium and observed under a fluorescence microscope.

### RNA-seq and bioinformatic analyses

Two pools of sorted PGCs from to 6–14 individuals of each sex and stage were used as templates. Since the yield of total RNA was very low, cDNA libraries were prepared using SMART-seq v4 Ultra Low Input RNA Kit for Sequencing (TaKaRa Bio), which is based on the SMART-seq2 method^58^. Then Nextera XT DNA Library Preparation Kit (Illumina Inc., San Diego, CA, USA) was used to make cDNA libraries suitable for Illumina sequencing. These were analyzed using the Hiseq system (Illumina Inc.) with 150 bp paired-end sequencing.

The quality of the sequencing results was assessed using FastQC (ver. 0.11.9). Trimming and quality filtering of the raw reads were performed using Trimmomatic (ver. 0.39)^59^ as well as Trim Galore (ver. 0.6.6) with Cutadapt (ver. 3.1)^60^ software. The trimmed and filtered reads were mapped to the chicken reference genome (GRCg6a) using STAR (ver. 2.7.3a)^61^. The uniquely mapped reads in each gene were counted using the featureCounts tool (ver. 2.0.1)^62^. Detection of sex-biased genes was performed using the DE-seq2 tool (ver. 1.20.0)^63^. MA-plots were created using the Bokeh visualization library (ver. 2.3.2). Then, GO and KEGG pathway analyses were performed using the g:Profiler online tool^64^. Finally, tissue or cell-specificity was characterized using the Metascape online tool^65^.

### RT-qPCR and RT-PCR

To confirm the results of RNA-seq, RT-qPCR analysis was conducted. In this analysis, whole transcriptome amplification was performed using the QuantiTect Whole Transcriptome Kit (QIAGEN, Venlo, Netherlands) against total RNA from new pools of sorted PGCs from 45–70 embryos of each stage and sex. To remove any bias in the whole transcriptome amplification reaction, the reaction was performed in triplicate for each stage and sex (*n* = 3). The synthesized cDNA was then used for RT-qPCR using a StepOne real-time PCR system (Applied Biosystems, Waltham, MA, USA) with the KOD SYBR qPCR Mix (Toyobo Co. Ltd., Osaka, Japan). For the stable PCR reaction, a total of 0–10% concentration of dimethyl sulfoxide was used. The primers used for this analysis are listed in supplementary Table S5. The RT-qPCR conditions were as follows: 40 cycles of 98 °C for 10 s, 60–65 °C for 10 s, and 68 °C for 30 s. Melting curve analysis was performed after this amplification stage in three steps, 95 °C for 15 s, 60 °C for 1 min, and 95 °C for 15 s.

Gene expression analysis of cultured PGCs stimulated with RA was also conducted using RT-qPCR. Total RNA was purified using the RNeasy Micro Kit (QIAGEN). For cDNA synthesis, SuperScript IV reverse transcriptase (Thermo Fisher Scientific) was used against 100 ng of total RNA. The RT-qPCR reaction was also performed using the StepOne real-time PCR system (Applied Biosystems) with the KOD SYBR qPCR Mix (Toyobo Co. Ltd.), and the primers used in this reaction are shown in supplementary Table S6. The RT-qPCR conditions were as follows: 40 cycles of 98 °C for 10 s, 60–64 °C for 10 s, and 68 °C for 30 s, followed by melt curve analysis under the same conditions described above.

Relative expression scores were calculated using the ΔΔCt method^66^. The scores of each target were normalized to those of GAPDH or *β*-actin.

RT-PCR was conducted to confirm the expression of the RA receptors, *RARα*, *RARβ*, and *RARγ*. RT-PCR was performed using KOD One® PCR Master Mix (Toyobo Co. Ltd.) under the following conditions: 35 cycles of 98 °C for ten seconds, 60 °C (*RARα*) or 64 °C (*RARβ*, and *RARγ*) for five seconds, and 68 °C for one second. Primers for *RARα* and *RARγ* amplification were designed in this study as follows: forward, 5′-AGACGGAGTGCTCGGAGAGT-3′ and reverse, 5′-GGCGAACTCCACCGTCTTGA-3′ (*RARα*), and forward, 5′-GTGCGCAATGACCGCAATAA-3′ and reverse, 5′-ACAGTGAGGGGAAGGTCTCC-3′ (*RARγ*). Conversely, to amplify the *RARβ* fragments, two primers were designed according to a previous study^67^: forward, 5′-GTGTCAGTGCTTGTGAGGGA-3′ and reverse, 5′-TGCAGTACTGGCAGCGATTT-3′. The cDNA obtained from the negative controls (described below) was used as a template.

### Cell culture and stimulation test

Male PGCs sorted from the gonads of each E6.5 embryo were cultured in the culture medium as described in our previous study^52^, with some modifications. Briefly, KnockOut DMEM (Thermo Fisher Scientific) was supplemented with 1% chicken serum (Thermo Fisher Scientific), 1 × B-27 Supplement Minus Vitamin A (Thermo Fisher Scientific), 2 mM GlutaMAX™ (Thermo Fisher Scientific), 1 × EmbryoMAX nucleosides (Merck, Darmstadt, Germany), 1 × MEM Non-Essential Amino Acids Solution (Thermo Fisher Scientific), 1 × antibiotic–antimycotic mixed stock solution (Nacalai Tesque, Kyoto, Japan), 0.5 mM monothioglycerol (FUJIFILM Wako Pure Chemical Co., Osaka, Japan), 1 × sodium pyruvate, 10 ng/mL human FGF2 (PeproTech Inc., Rocky Hill, NJ, USA), 1 unit/mL heparin (Merck), 0.2 μM H1152 (FUJIFILM Wako Pure Chemical Co.,), and 0.2 μM Blebbistatin (FUJIFILM Wako Pure Chemical Co.,). The sorted PGCs were cultured at 38 °C with 5% CO_2_ and 3% O_2_ and subcultured every 2–3 days. Cultured PGCs were observed using an inverted microscope (IX71; Olympus) and photographed with an Olympus DP70 camera (Olympus).

Since the number of PGCs isolated from male E6.5 gonads was very low, the PGCs were cultured and proliferated for approximately two months. These were then used for stimulation tests using RA. In this stimulation test, PGCs derived from five individuals and cultured independently were used (*n* = 5). Male PGCs, which were seeded at 2.5 × 10^4^ cells in a 35-mm dish and cultured for 24 h, were stimulated with all-trans-Retinoic acid (R2625; Merck) at a dose of 1 or 10 μM for 24 h. To prepare the RA-containing PGC medium, RA powder was first dissolved in 99.5% EtOH at a concentration of 5 mM. The RA solution was then diluted with culture medium. As a negative control, the cultured PGCs were stimulated with EtOH.

### Statistical analysis

Statistical analyses for the RT-qPCR experiments were performed using Dunnett's test (two-tailed) to evaluate the differences between the negative controls and RA-stimulated samples using R software (ver. 3.6.3). *P* < 0.05 was considered statistically significant.

## Supplementary Information

Supplementary Information 1.

Supplementary Information 2.

## Data Availability

All RNA-seq datasets are publicly available at Gene Expression Omnibus under accession number GSE188689.
