# Supplementary material for: Prediction of sex-determination mechanisms in avian primordial germ cells using RNA-seq analysis
Source: Sci Rep. 2022 Aug 17;12:13528. doi: 10.1038/s41598-022-17726-7 (PMC9385715; doi:10.1038/s41598-022-17726-7)
Supplement: Supplementary file 1 — Supplementary Information 1. [file 41598_2022_17726_MOESM1_ESM.pdf]

Prediction of sex-determination mechanisms in avian primordial germ cells using RNA-seq analysis

Kennosuke Ichikawa[1*], Yoshiaki Nakamura[2], Hidemasa Bono[2], Ryo Ezaki[2], Mei Matsuzaki[2], & Hiroyuki Horiuchi[1,2]

[1]Genome Editing Innovation Center, Hiroshima University, 3-10-23 Kagamiyama, Higashi-Hiroshima, Hiroshima 739-0046, Japan

[2]Graduate School of Integrated Sciences for Life, Hiroshima University, 1-4-4 Kagamiyama, Higashi-Hiroshima, Hiroshima 739-8528, Japan

[*]Corresponding author: Kennosuke Ichikawa

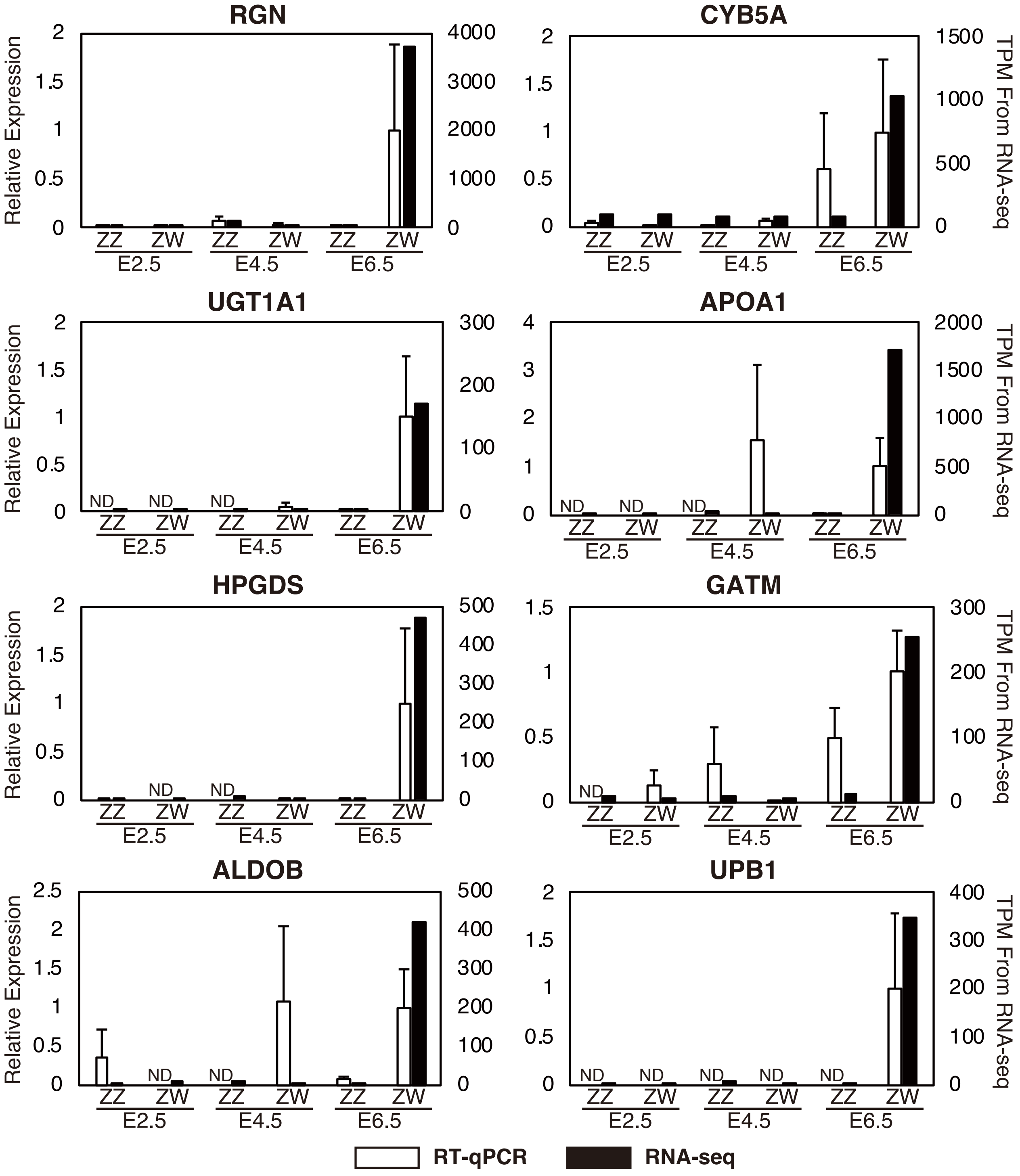

Supplementary Figure S1. Expression analysis of the female-biased genes detected in E6.5 chicken embryos using RT-qPCR. The left y-axis indicates the score of RT-qPCR. The right y-axis indicates the average scores of TPM from two biological replicates. Open and closed bars indicate the results of RT-qPCR and RNA-seq, respectively. The 2-ΔΔCt method was used for the calculation of relative expression levels, which were normalized using levels of GAPDH. Error bars indicate SE of triplicates (n = 3). ND means not detected.

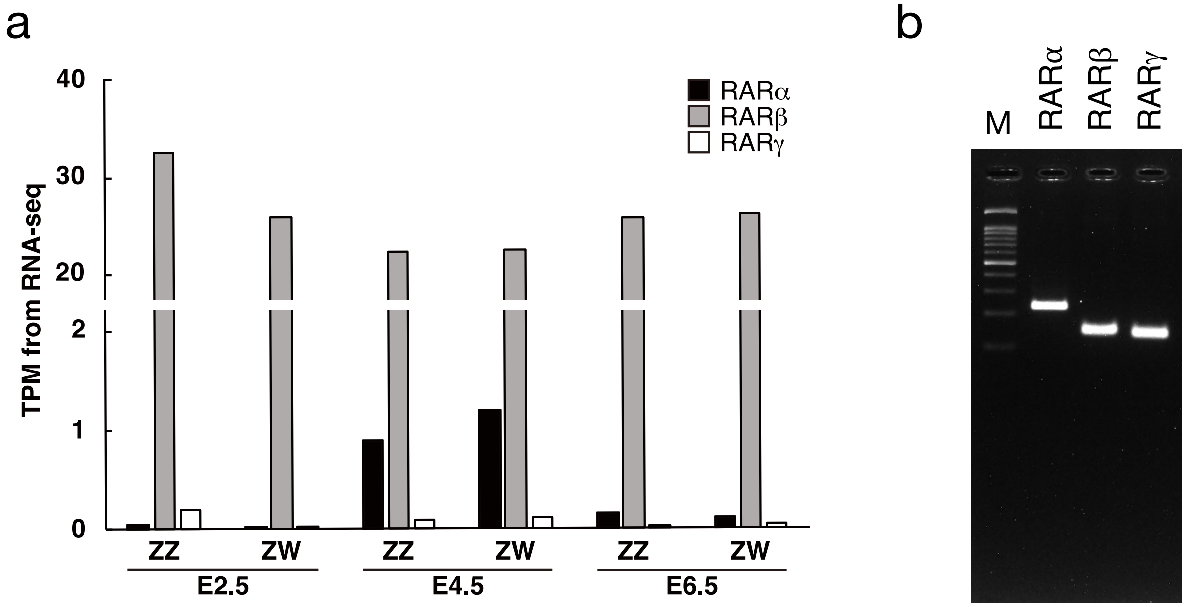

Supplementary Figure S2. Evaluation of the expressions of Retinoic acid receptors (RARs). (a) The expression levels of RARα, RARβ, and RARγ, determined by RNA-seq analysis in TPM units in each sex and developmental stage. (b) Gel electrophoresis of the RT-PCR fragments. The predicted sizes of the fragments are 217, 129, and 133 bp, respectively. M, 100 bp marker.

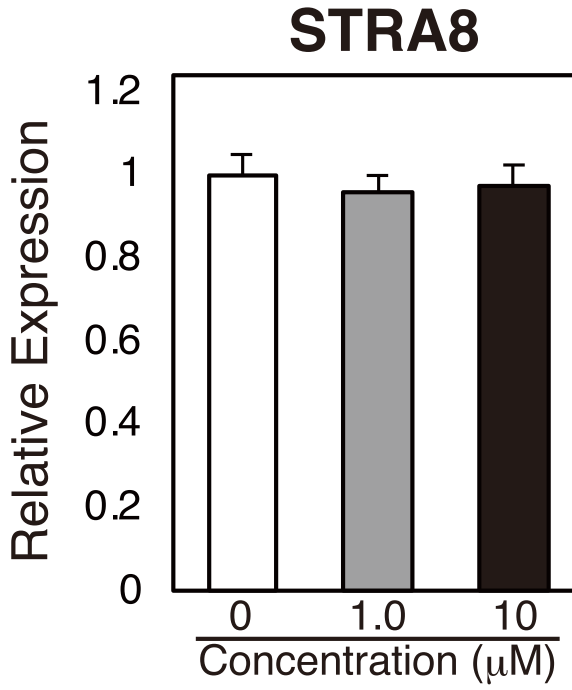

Supplementary Figure S3. Expression analysis of *STRA8* under RA stimulation. The x-axis indicates the concentration of RA. The $2^{-\Delta\Delta Ct}$ method was used for the calculation of relative expression levels, which were normalized using expression levels of *β-actin*. Error bars indicate SE of the mean of relative expression levels in independently cultured gPGCs derived from five individuals (n = 5).
